# William C. Spooner, M.R.C.V.S.

**Published:** 1885-07

**Authors:** 


### OBITUARY.

### WILLIAM C. SPOONER, M.R.C.V.S.

Veterinarians have lost an old and valuable associate in Mr. Spooner, who died at Eling, near Southampton, May 3, in the 76th year of his age. He was known in this country chiefly through a work on the horse by Youatt & Spooner," which was at one time a standard authority. His other works, "Cattle Medicine," "Compendium of the Veterinary Art," and "Sheep; their History, Anatomy and Diseases," passed through several editions. During the latter part of his life he devoted himself exclusively to agricultural pursuits.

